# Physiologic oxygen responses to smoking opioids: an observational study using continuous pulse oximetry at overdose prevention services in British Columbia, Canada

**DOI:** 10.1186/s12954-024-01011-z

**Published:** 2024-05-03

**Authors:** Jessica Moe, Jane A. Buxton, Yueqiao Elle Wang, Tamara Chavez, Damian Feldman-Kiss, Charotte Marr, Roy A. Purssell, Michael Otterstatter

**Affiliations:** 1 Department of Emergency Medicine, University of British Columbia, Diamond Health Care Centre, 11th Floor – 2775 Laurel Street, Vancouver, BC V5Z 1M9 Canada; 2 BC Centre for Disease Control, 655 West 12th Avenue, Vancouver, BC V5Z 4R4 Canada; 3 School of Population of Public Health, University of British Columbia, 2206 East Mall, V6T 1Z8 Vancouver, BC Canada; 4 CoVaRR-Net’s Indigenous Engagement, Development, and Research Pillar 7, University of British Columbia, 103-1690 Nelson Street, Vancouver, BC V6G 1M5 Canada; 5 Portland Hotel Society, 9 East Hastings Street, Vancouver, BC V6A 1M9 Canada

**Keywords:** Pulse oximetry, Oxygen saturation, Smoking, Opioid overdose, Harm reduction

## Abstract

**Background:**

In British Columbia, Canada, smoking is the most common modality of drug use among people who die of opioid toxicity. We aimed to assess oxygen saturation (SpO_2_) while people smoked opioids during a pilot study that introduced continuous pulse oximetry at overdose prevention services (OPS) sites.

**Methods:**

This was an observational cohort study, using a participatory design. We implemented our monitoring protocol from March to August 2021 at four OPS. We included adults (≥ 18 years) presenting to smoke opioids. A sensor taped to participants’ fingers transmitted real-time SpO_2_ readings to a remote monitor viewed by OPS staff. Peer researchers collected baseline data and observed the timing of participants’ inhalations. We analyzed SpO_2_ on a per-event basis. In mixed-effects logistic regression models, drop in minimum SpO_2_ ≤ 90% in the current minute was our main outcome variable. Inhalation in that same minute was our main predictor. We also examined inhalation in the previous minute, cumulative inhalations, inhalation rate, demographics, co-morbidities, and substance use variables.

**Results:**

We recorded 599 smoking events; 72.8% (436/599) had analyzable SpO_2_ data. Participants’ mean age was 38.6 years (SD 11.3 years) and 73.1% were male. SpO_2_ was highly variable within and between individuals. Drop in SpO_2_ ≤ 90% was not significantly associated with inhalation in that same minute (OR: 1.2 [0.8–1.78], *p* = 0.261) or inhalation rate (OR 0.47 [0.20–1.10], *p* = 0.082). There was an association of SpO_2_ drop with six cumulative inhalations (OR 3.38 [1.04–11.03], *p* = 0.043); this was not maintained ≥ 7 inhalations. Demographics, co-morbidities, and drug use variables were non-contributory.

**Conclusions:**

Continuous pulse oximetry SpO_2_ monitoring is a safe adjunct to monitoring people who smoke opioids at OPS. Our data reflect challenges of real-world monitoring, indicating that greater supports are needed for frontline responders at OPS. Inconsistent association between inhalations and SpO_2_ suggests that complex factors (e.g., inhalation depth/duration, opioid tolerance, drug use setting) contribute to hypoxemia and overdose risk while people smoke opioids.

**Supplementary Information:**

The online version contains supplementary material available at 10.1186/s12954-024-01011-z.

## Background

The opioid overdose epidemic continues to claim a disproportionate number of lives internationally. Worldwide between 2010 and 2019, the prevalence of opioid use increased by 76% and the estimated number of people who used opioids doubled. Globally, 61 million people used opioids in 2020 [1]. Persistently high opioid poisoning deaths in North America have been driven by fentanyl and its analogues [1, 2] in the unregulated drug supply. In Canada, there were 3,970 apparent opioid toxicity deaths from January to June 2023 (22 deaths per day), which represented a 5% increase from the same period in 2022.

British Columbia (BC) is one of the hardest hit provinces in Canada [2] with 43.1 unregulated drug toxicity deaths per 100,000 population [3]. In BC, smoking has become the most common mode of non-medical drug use since 2017 [4] and is currently the most common modality of use among people who die of drug toxicity: from 2016 to 2020, non-medical drug toxicity deaths due to smoking increased from 31 to 56%, while deaths attributable to injection declined from 39 to 19% [5]. Similar trends towards rising unregulated opioid use via smoking and declining use via injection have been noted in the United States [6], and across Europe, where 51% of clients entering drug treatment facilities for heroin use in 2021 reported smoking or inhaling their opioids [7]. Qualitative explorations suggest that people’s preference for smoking opioids is driven by desire for safer use [8], as many perceive smoking to be associated with a lower overdose risk [6, 8]. However, hypoxemia is an expected pathophysiologic occurrence with opioid inhalation. One study showed that hypoxemia developed equally among people who used heroin by inhalational and intravenous routes (12.5% experienced drops in oxygen saturation), regardless of modality of use [9]. Based on limited available evidence, onset of action would be expected to be comparable and rapid after both smoking and injecting. In a study involving two volunteer subjects, heroin appeared rapidly in the blood and peaked within 1–5 min following smoking, similar to when administered intravenously [10]. Furthermore, fentanyl, which was implicated in 82% of drug toxicity deaths in BC in 2022 [11], has a rapid onset of action regardless of whether it is consumed intravenously or by inhalation [12]. We would therefore expect that blood levels of fentanyl would rise even quicker, and cause more rapid hypoxemia, than heroin when smoked. Still, people who smoke opioids (predominantly unregulated fentanyl in many jurisdictions) may be less likely to carry a naloxone kit [13], to use overdose prevention services sites, and more likely to use drugs alone [14]. Increased drug toxicity mortality related to smoking also reflects barriers to smoking-specific harm reduction services, including regulatory restrictions (as of May 2023, only 2 of 38 federally sanctioned supervised consumption sites in Canada were authorized to provide inhalation services) [15] and space constraints (e.g., inadequate ventilation and/or poorly monitored, secluded spaces) [16].

Overdose prevention services (OPS), sanctioned under Ministerial Order in response to the overdose death-related public health emergency in BC, allow supervised drug consumption and overdose response without federal exemption and therefore can be established more quickly than supervised consumption sites [17]. Specifically in BC, as of December 2023, 22 of 50 OPS or supervised consumption sites permitted inhalation [18], therefore offering a unique opportunity to develop, implement, and evaluate interventions to improve smoking-specific harm reduction services in BC. Supervised consumption sites and drug consumption rooms exist in many countries, including Australia, Germany, Switzerland, the Netherlands, Spain, Portugal, and France [19]. In the United States, supervised consumption sites are operational in New York City and Rhode Island [20]. A review of 39 drug consumption facilities in six European countries indicated that on average, sites offered 7–8 places for injection and 6–7 places for smoking/inhalation. At 30 sites in the Netherlands, where smoking is more prevalent, sites offered an average of 14 places for smoking and 5 places for injection drug use [21]. Therefore, there is a global imperative to develop guidance regarding optimized monitoring for people using drugs via inhalation at supervised drug consumption sites. In addition to overdose prevention, benefits [22] of providing safer smoking spaces include decreasing infectious disease transmission [23, 24], minimizing exposure to violence, social networking and support [25], reducing harms resulting from “rushed” smoking [26], and engaging people who smoke in harm reduction services [27]. There is a critical need to develop services that specifically mitigate smoking-related risks, in order to curb a concerning rise in smoking-related drug toxicity deaths.

Opioid administration has been associated with hypoxemia in animal models [28] and human patients in controlled settings [29, 30]. Oxygen level responses in patients with opioid use disorder have been assessed in a limited number of patients receiving injected opioids under medical supervision. In one study examining a single subject on long-term pharmaceutical diacetylmorphine (heroin) treatment, injections caused apnea (maximum 56 s) and hypoxemia (minimum oxygen saturation [SpO_2_] 80%). The authors found a general dose-response effect in respiratory depression related to injection, measured by hypoxemia, hypercarbia, and depressed respiratory rate. However, the relationship was inconsistent; apnea also occurred at lower diacetylmorphine doses [31]. Another study found that 4 of 10 patients administered injectable diacetylmorphine or methadone experienced a drop in SpO_2_ < 90% for > 10 s; however, correlation with other measures of respiratory depression was inconsistent [32]. To our knowledge, no studies to-date have evaluated physiologic oxygen responses to smoking unregulated opioids in real-world settings. An understanding of how SpO_2_ responds to smoking non-medical opioids as they are used in real world settings is critical, particularly in the context of dynamic unregulated drug supplies and changing patterns of drug use.

While smoking-related drug toxicity deaths increase, risks associated with smoking opioids are poorly understood: it is not known how and when peoples’ oxygen levels change while smoking. Understanding dynamics of hypoxemia development is a foundational step to developing evidence-informed messaging regarding overdose risks of smoking opioids and optimized monitoring and response mechanisms (e.g., phone apps) that reflect actual physiologic responses to smoking. Tailored education and monitoring could decrease risk for people smoking alone or in other high-risk situations. The objective of this study was to determine if number of inhalations while people smoke opioids is associated with decreases in SpO_2_. It was undertaken during a pilot project that introduced continuous pulse oximetry at OPS sites. We hypothesized that number of inhalations would be cumulatively associated with drops in SpO_2_ while people smoked opioids.

## Methods

### Study setting

We developed and implemented a novel, continuous pulse oximetry monitoring protocol (i.e. continuous, real-time monitoring of clients’ oxygen levels and heart rate for the duration of their observed drug use) from March to August 2021 at four health authority-approved, non-profit, peer-staffed OPS in BC with indoor and outdoor smoking facilities: Overdose Prevention Society in Vancouver’s Downtown Eastside; Rock Bay Landing (also providing emergency and transitional housing), operated by Victoria Cool Aid Society; Travelodge (a residential site), operated by AIDS Vancouver Island; and SOLID Outreach Society (peer-based health education and harm reduction services) in Victoria.

### Population

We included adults (≥ 18 years) presenting to OPS to smoke opioids. Participants received a $20 honorarium and partnering OPS received $10 per enrollment to compensate for their time and assistance [33]. Participants could enroll once daily and multiple times on different days.

### Study design

This was an observational cohort study. We describe our participatory design, implementation, and the feasibility and acceptability of our protocol in a parallel study [34, 35]. People with lived/living experience of substance use were involved throughout study planning, implementation, and analysis. We recruited and trained peer researchers to implement processes, assist staff to apply continuous pulse oximetry, recruit and consent participants, and collect data.

**Fig. 1:**
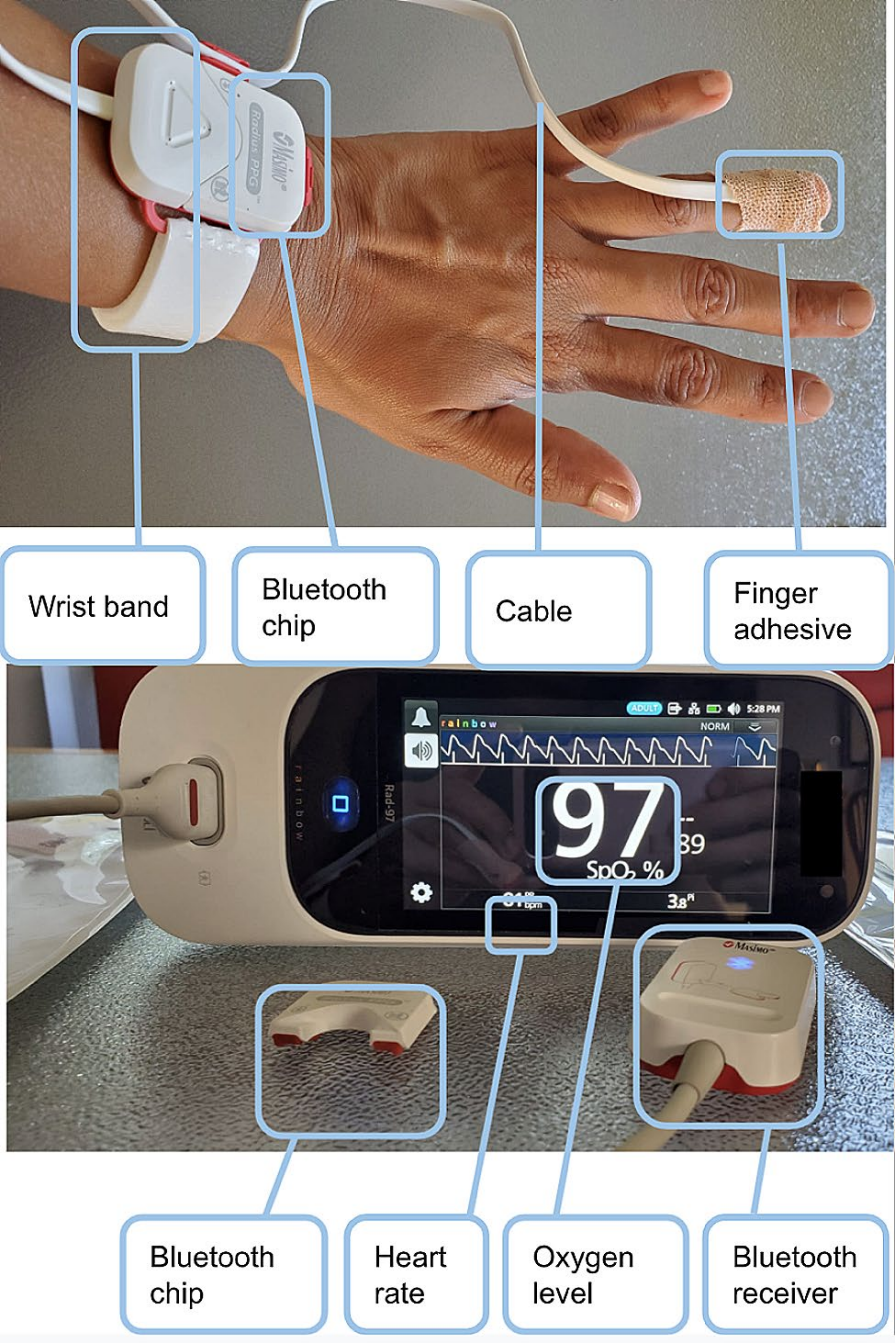
Continuous pulse oximetry sensors and monitors

Continuous pulse oximetry was only available to study participants at participating OPS. We purchased continuous pulse oximetry devices from a manufacturer that supplied similar monitoring devices at local hospitals. The devices included a sensor taped to participants’ fingers that transmitted real-time SpO_2_ data to a remote monitor via Bluetooth for OPS staff to view (Fig. 1) [34, 35].

Sensors and monitors were applied for the duration of clients’ monitored drug use, with no specified minimum or maximum time that the devices were applied. Participants used drugs as they normally would, and were permitted to talk. Participants often conversed with other clients in their vicinity, but in other circumstances (particularly if other clients were not present) stayed generally still, choosing to sit quietly while using their drugs. An alarm sounded if SpO_2_ decreased to ≤ 90% for 15 s, based on evidence that arterial oxygen content and therefore oxygen delivery to tissues steeply declines once oxygen saturation falls below 90%, due to a steepening decline (the “slippery slope”) on the oxygen-hemoglobin dissociation curve [36]. The 15 s duration of hypoxemia prior to alarms sounding was based on prespecified manufacturer settings for the monitors used during this study, to balance sensitivity and specificity of alarms. For each monitoring event, SpO_2_ and heart rate readings were recorded in oximetry software, accurate to the second.

Prior to participating in the continuous pulse oximetry protocol, peer researchers collected data from participants using a standardized data collection form (Appendix 1). Peer researchers conducted structured observations of enrolled participants while they smoked opioids and OPS staff while they monitored participants. Using a standardized form (Appendix 2) and a digital stopwatch, peer researchers marked times that participants inhaled their drugs, as well as times of alarms, and OPS staff interventions and responses (accurate to the minute).

### Oxygen saturation data aggregation

**Fig. 2:**
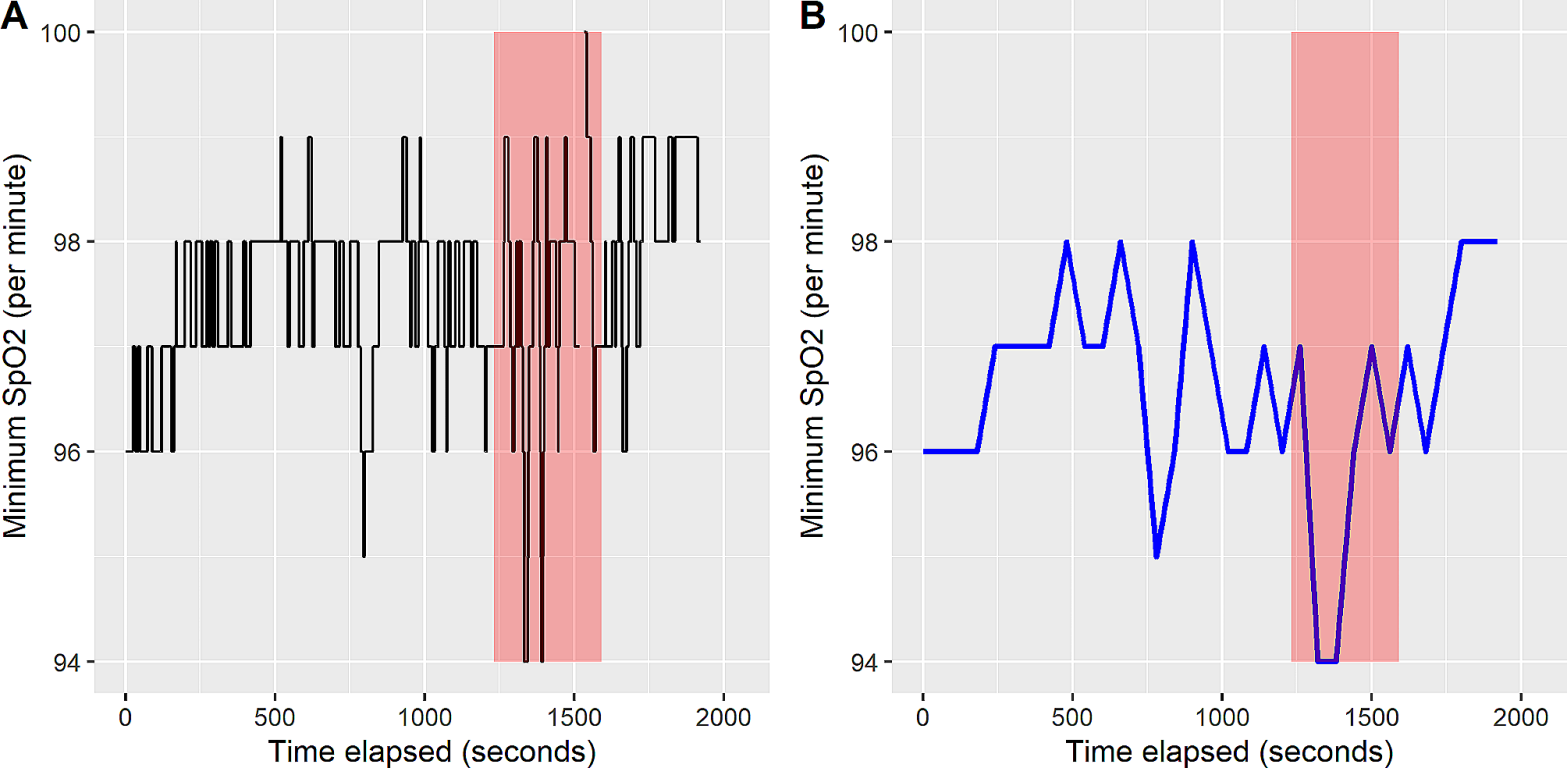
The left panel (**A**) shows the raw per second SpO_2_ data (solid black line) from a single client with inhalation times highlighted in red. The right panel (**B**) shows the same data aggregated on a per minute basis. Inhalation times (on per minute scale) are shown as vertical red lines

While SpO_2_ data was recorded on a per second basis, inhalations were recorded on a per minute basis, and therefore the exact (per second) timing of each inhalation was unknown and not recorded. To analyze SpO_2_ and inhalation data together, for each client, we calculated the per minute mean, minimum, and standard deviation of SpO_2_ from raw data. We then identified inhalation timepoints in the aggregate (per minute) data. See Fig. 2 for illustration.

### Statistical methods

For modeling purposes, we used per minute aggregate data for each individual’s monitoring session. We analyzed the tracings on a per-event basis, as we sought to understand associations between factors related to both the individual (e.g., co-morbidities) and their drug use patterns (e.g., inhalations) and SpO_2_ responses during each drug use event. In the analysis model, we removed SpO_2_ data that extended beyond 50 min for each monitoring event, given our on-the-ground experience, captured in structured observations, that monitoring events were generally of shorter duration (median 13.5 [IQR: 8.3, 23.8] minutes), with only 22 of 599 participants monitored for more than 50 min.

**Fig. 3:**
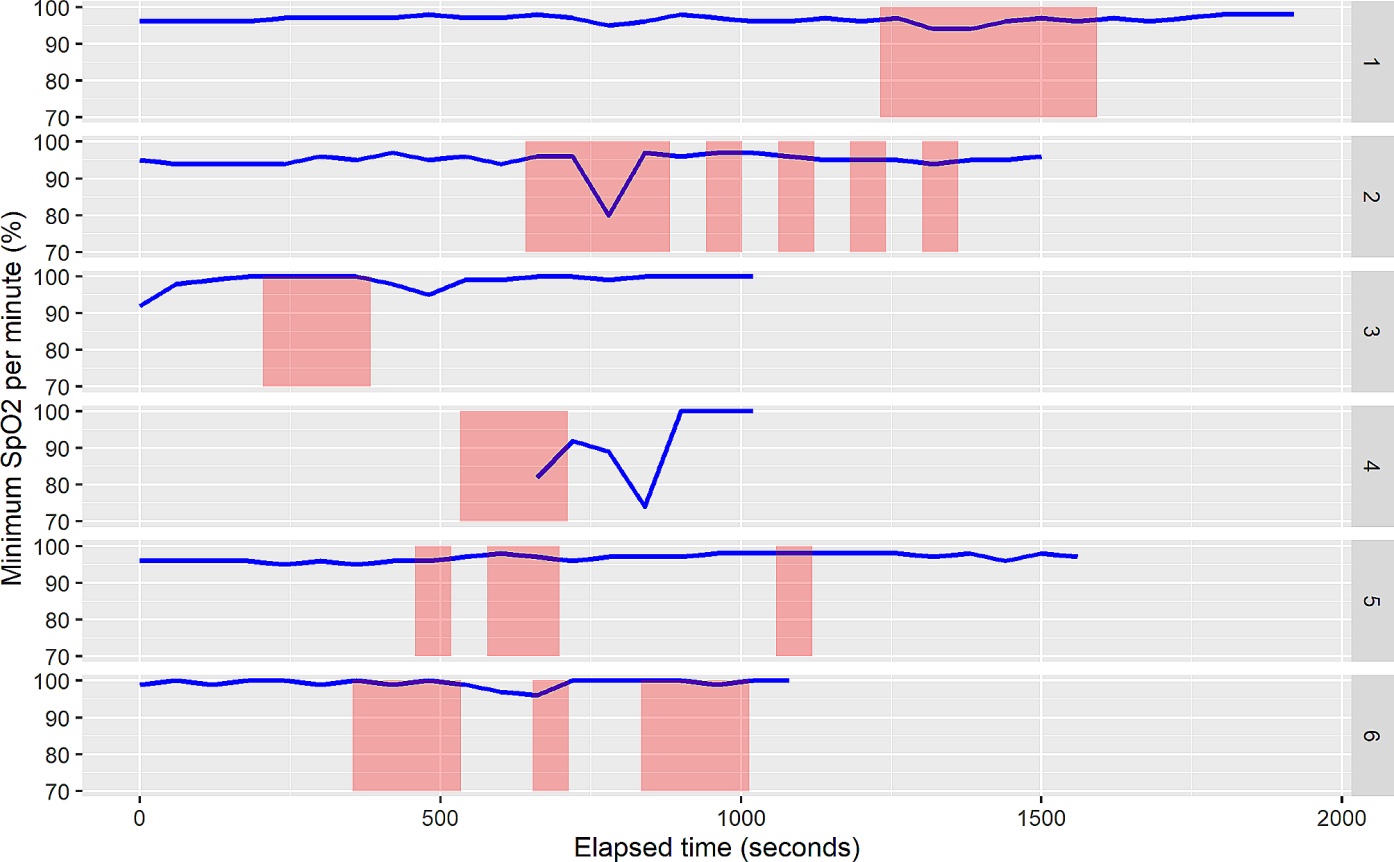
The first six clients in our analysis dataset, with per minute aggregate data as described above. The y-axis is the per-minute minimum SpO_2_ value and red lines indicate minutes with inhalations. Larger red areas indicate several consecutive inhalations. Occasionally, SpO_2_ was recorded only intermittently resulting in gaps or incomplete data (e.g., Panel 4)

We used a mixed-effects logistic regression modelling framework, allowing for proper accounting of the repeated (per minute) observations on individuals. In these models, drop in minimum SpO_2_ to 90% or less (yes or no) in the current minute was our main outcome variable and inhalation in that same minute (yes or no) was treated as our main predictor variable. Occasionally, SpO_2_ was recorded only intermittently resulting in gaps in the SpO_2_ data. See Fig. 3 for illustration.

We also examined inhalation in the previous minute (yes or no), cumulative number of inhalations recorded (range, approximately 1 to 10), and inhalation rate (number of inhalations / time elapsed) over the entire observation period until the minute in question as alternative predictor variables. The individual client ID was included as a random effect term to account for correlations among observations on the same individual. We included log-transformed elapsed time as a covariable for each individual (i.e., as an offset term), to account for differing amounts of time under observation for each client. These analyses excluded periods without SpO_2_ data.

### Covariables

In addition to inhalation, we examined available demographic (e.g., gender, age), medical (e.g., reported co-morbidities), and substance use variables (e.g., substances participants believed they were smoking), as covariables in our analysis (described below). Events with absent or invalid data were eliminated from the analyses; we did not perform imputation for missing values. Initial analyses showed no significant association between demographics, medical co-morbidities, or substance use variables and the outcome, so these variables were removed from further analyses. The analysis was not pre-registered, and the results should be considered exploratory.

## Results

**Fig. 4:**
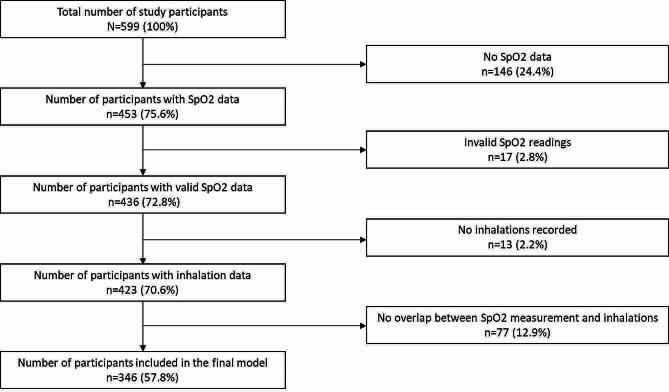
Study flowchart

In total, 599 smoking events occurred during the study period. We excluded participants who had either absent (146/599, 24.4%) or invalid SpO_2_ readings (17/599, 2.8%), leaving 72.8% (436/599) of our original cohort who had analyzable SpO_2_ data. Additionally, we removed events with no recorded inhalations (13/599, 2.2%), and clients with no overlap between inhalation and SpO_2_ data (77/599, 12.9%). Following these exclusions, we included 346 of 599 original participants (57.8%) in our analysis. See Study Flowchart (Fig. 4) for full breakdown of study exclusions.

### Characteristics of study cohort

The characteristics of our study cohort are shown in Table 1.

**Table 1:** Self-reported characteristics of the full study cohort

Characteristic	Study cohort (*N* = 599)
**Age (mean ± SD)**	38.6 ± 11.3 *(26 missing data)*
**Gender, N (%)**	
Male	438 (73.1%)
Female	148 (24.9%)
Other*	6 (0.9%)
Prefer not to answer	2 (0.3%)
Missing	5 (0.8%)
**Race, N (%)**	
White	406 (67.8%)
Indigenous or Metis	136 (22.7%)
Mixed	25 (4.2%)
African-American or Asian	9 (1.5%)
Prefer not to answer	10 (1.7%)
Missing	13 (2.2%)
**Employment, N (%)**	
Unemployed	348 (58.1%)
Part-time	86 (14.4%)
Paid volunteer	82 (13.7%)
Full-time	22 (3.7%)
Prefer not to answer	45 (7.5%)
Missing	16 (2.7%)
**Housing status, N (%)**	
Supported housing	187 (31.2%)
Shelter	149 (24.9%)
On the street	86 (14.4%)
Single room occupancy hotel	81 (13.5%)
Own house/apartment	32 (5.3%)
Couch surfing/friends/family	29 (4.8%)
Other**	14 (2.3%)
Prefer not to answer	11 (1.8%)
Missing	10 (1.7%)
Comorbidities, N (%)***	
None	354 (59.1%)
Mental health	161 (26.9%)
Pulmonary	71 (11.9%)
Cardiac	27 (4.5%)
Infectious disease	16 (2.7%)
Diabetes	11 (1.8%)
Other****	9 (1.5%)
Prefer not to answer	17 (2.8%)
**Had ever previously overdosed while smoking opioids, N (%)**	
Yes	265 (44.2%)
No	215 (35.9%)
Unknown	119 (19.9%)

*Other genders included transgender, two-spirit, and gender-nonconforming

**Other housing included hospital, tent, sailboat, and a combination of accommodations

***n = 55 participants reported more than one comorbidity

****Other disorders included chronic pain, neuromuscular, renal, and rheumatologic disorders

In our full study cohort (*N* = 599), participants had a mean age of 38.6 years (standard deviation 11.3 years). Most participants were male (73.1%) and 67.8% were White. Participants were recruited from our sites as follows: Overdose Prevention Society in Vancouver BC (*n* = 93), and Rock Bay Landing (*n* = 91), AIDS Vancouver Island (*n* = 185), and SOLID Outreach Society (*n* = 230) in Victoria, BC. Over half (58.1%) reported being currently unemployed. Most often participants reported living in either a shelter (24.9%) or supported housing (31.2%). Mental health conditions were the most commonly reported comorbidities (26.9%). Overall, 44.2% (265/599) of participants reported that they had ever previously overdosed while smoking opioids. This proportion was similar in our analysis dataset (42.2%, 146/346).

### Critical incidents

There were 77 instances in our SpO_2_ data that met our *a priori* alarm threshold of an SpO_2_ level ≤ 90% lasting for 15 s or longer.

During all monitoring events, there were no reported instances of overdose. Recorded interventions were limited to one episode of verbal and physical stimulation. There were no recorded interventions of naloxone, oxygen, rescue breathing, chest compressions, airway support, defibrillation, epinephrine, or ambulance calls. None of our included participants were transported to hospital during the study period.

### Variability among monitoring events

Duration of continuous pulse oximetry monitoring events ranged from 0.2 to 308.4 min. Number of inhalations per session ranged from 1 to 10. Overall, our SpO_2_ data demonstrated marked heterogeneity. Among individual recorded monitoring events, mean SpO_2_ ranged from 74.6 to 100%, maximum SpO_2_ ranged from 79 to 100%, and minimum levels were highly heterogeneous with transient fluctuations assumed to be measurement error (e.g., non-sustained SpO_2_ = 0%).

The SpO_2_ data showed a high degree of variability both within and between individuals, and were not normally distributed (Kolmogorov-Smirnov [K-S] normality test *p* < 0.05). The skewness (-8.2) indicated left-tailed outliers.

### Association between inhalations and oxygen saturation

**Fig. 5:**
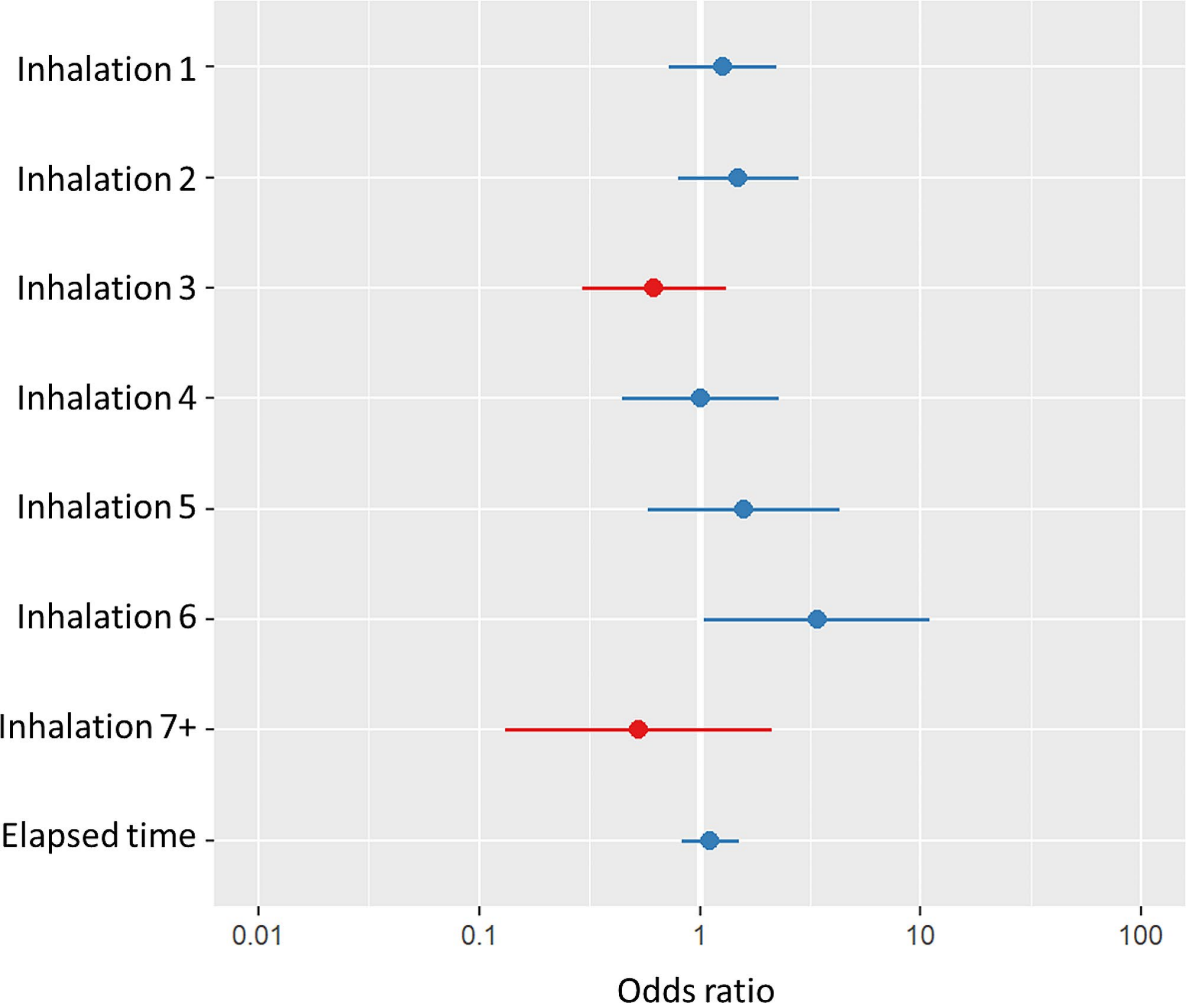
Association between cumulative number of inhalations and the odds ratio for drop in SpO_2_

Our analysis did not suggest a straightforward effect of inhalation on SpO_2_. Drop in SpO_2_ ≤ 90% in the current minute was not significantly associated with inhalation in that same minute (OR 1.2 [95% CI: 0.86–1.78], *p* = 0.261) or the inhalation rate prior to the minute in question (OR 0.47 [95% CI: 0.20–1.10], *p* = 0.082). However, the analysis suggested a possible dose-response effect of inhalation on SpO_2_, as the cumulative number of inhalations was associated with an increasing probability of SpO_2_ drop. By the sixth inhalation, the likelihood of a drop in SpO_2_ was significant (OR 3.38 [95% CI: 1.04–11.03], *p* = 0.043), but this trend was not maintained for seven or more inhalations (Fig. 5).

We did not find a clear effect of self-reported substance use on the SpO_2_. Regardless of the substances that clients believed they were currently smoking (either exclusively opioids, or opioids in combination with other substances), average SpO_2_ was similar (mean ± SD: heroin/fentanyl alone: 97.1% ± 2.6%; opioids and stimulants: 97.5% ± 1.3%; opioids and other substances: 96.9% ± 3.2%) and there was no significant effect on the probability of a drop in SpO_2_ (heroin/fentanyl alone: OR 0.81 [95% CI: 0.25–2.55], *p* = 0.714; opioids and stimulants: OR 0.06 [95% CI: 0.00–1.54], *p* = 0.089; opioids and other substances: OR 0.91 [95% CI: 0.40–2.11], *p* = 0.833).

## Discussion

Our study supports that continuous pulse oximetry, by making SpO_2_ data available to OPS staff to view in real-time, is a safe adjunct for monitoring people who smoke opioids at OPS. In addition to SpO_2_ data presented in this study, in our companion feasibility analysis, OPS staff and clients identified that remote monitoring imparted feelings of safety, comfort, confidence, and allowed observation from afar to prevent exposure to respiratory infections and COVID-19 [34, 35]. Our study also demonstrates real challenges of implementing SpO_2_ monitoring and community-driven research in the dynamic, real-world setting of OPS. Only 72.8% (436/599) of our original cohort had valid, analyzable SpO_2_ data and our analytic cohort comprised only 57.8% (346/599) of participants who had valid overlapping oxygen and inhalation data. Unusable and inconsistent SpO_2_ data reflects unique barriers to obtaining accurate SpO_2_ readings in our study setting. Accurate SpO_2_ readings were dependent on multiple complicating factors including correct sensor position on clients’ fingers (peer researchers were trained to reposition sensors if they were not receiving a consistent reading), changing environmental conditions (e.g., cool digits in cold weather), inconsistent individual optimization for monitoring when recruited in the community (e.g., dehydration leading to decreased finger perfusion; paint or soil on nails), and variable spaces (e.g., difficulty maintaining connections when the monitor was at greater distances from the individual) [34]. Despite being a very practical respiratory tool, continuous pulse oximetry is also a delayed peripheral measure. Therefore, alternative tools for measuring respiratory function and depression, such as capnography, could be considered and tested as an adjunct to this work. Moreover, inconsistent data could reflect inaccurate documentation of inhalation and critical incident data by peer researchers, signifying the importance of training, capacity building, and ongoing quality assurance in research conducted in community settings. We used drops in SpO_2_ to 90% or below as an outcome variable. Although we considered using drops relative to individuals’ baseline SpO_2_ as an outcome variable, as is commonly examined in the sleep literature [37, 38], individuals in our dataset had inconsistent baseline SpO_2_ data, limiting our ability to examine relative drops. Other studies could integrate baseline monitoring into individuals’ observation periods to allow relative drops to be quantified.

Our study demonstrates that individuals’ SpO_2_ respond variably to smoking opioids. Among all recorded events, mean SpO_2_ levels ranged from 74.6 to 100%, maximum levels from 79 to 100%, and minimum levels were highly variable with outliers that we assumed to be reading errors (e.g., transient SpO_2_ readings of 0%). Our data during monitoring in the observed OPS setting did not demonstrate a clear dose-response relationship between inhalations and hypoxemia. However, there was a signal towards an increasing likelihood of SpO_2_ drop with increasing number of inhalations, especially when an individual had inhaled 6 or more times. Although our results showed a weak association between number of inhalations and hypoxemia, based on existing literature [9, 10], we would intuitively postulate that episodes of desaturation in our study population could be associated with opioid inhalation over time and that the lack of statistical significance of our results was influenced by multiple complex factors in our uncontrolled study setting. Our findings corroborate the complex association found in previous studies, complicated even further by the variability of drug use in our study setting, compared to administration of known opioid doses by intravenous route in previous studies’ controlled clinical settings [31, 32]. Our findings indicate the complexity of titrating doses via smoking: the ultimate opioid dose and rate at which it is received is multifactorial, influenced by number, frequency, depth, and duration of inhalations. Due to these complex variables, we could not ensure consistency between inhalations and therefore expect that each inhalation delivered a variable dose of opioid, both within and between individuals. Our failure to detect a clear association between inhalations and SpO_2_ drop likely reflects that our analysis did not collect and capture important covariables of interest related to inhalation quality. Additionally, since people smoked their opioids at their own discretion, there could have been a selection bias where individuals who made seven or more inhalations had greater opioid tolerance and were therefore less likely to experience resultant hypoxemia. Our data also did not show a clear effect of suspected type of drug use and oxygen response, which in part reflects the inconsistency of a toxic, unregulated drug supply. Actual versus suspected drugs ingested, doses received, contaminants, and co-ingestants likely vary greatly (e.g., opioid contamination with other depressant drugs). Our findings have important implications in the context of a dynamic unregulated opioid supply recently characterized by increasing contamination with benzodiazepines, which may cause an additive effect on hypoxemia and apnea [39] and often a disproportionate effect on sedation [40]. In this context, having the capacity to continuously observe oxygen levels of clients experiencing prolonged sedation outlasting respiratory depression (e.g., after naloxone has reversed apnea but an individual remains drowsy) could allow OPS to safely monitor clients until their level of consciousness recovers. Reassuring oxygen levels in such situations could allow peer responders to avoid administering naloxone where not indicated, and could allow OPS to avoid activating emergency health service transport to hospital for people who remain drowsy but who are continuing to breathe independently and are oxygenating adequately.

Public health data clearly indicate an increase in fatal drug toxicity while smoking; however, our data have been unable to elaborate the characteristics of individuals or drug use that contribute to overdose. This suggests that additional variables not captured in our observational data contribute to risk. These may include difficult-to-measure factors, such as feeling rushed while using drugs in an environment prohibiting drug use, a context much different from using drugs in an authorized OPS setting among peers.

### Limitations

Our study was limited by having recorded inhalations on a per-minute basis, limiting our ability to understand the exact relation of inhalation timing to desaturations, which we defined based on 15-second epochs. We relied on peer researchers to collect data on inhalation timing while observing clients from a distance. In future extensions of our study, we suggest training these peers to record inhalations based on smaller time epochs. Validation work would be necessary to determine the exact time epochs that would be feasible to record in order to ensure a balance of granular data collection while maintaining accuracy, given the challenges of collecting data in the real-world setting of our study. Our inability to determine a clear association between smoking and SpO_2_ response was influenced by inter-event variability in participants, drug type, and drug use. Our failure to capture important potential covariables, such as quality of inhalations, participants’ baseline opioid tolerance, and actual type and dose of drug ingested, limited our analysis. Nonetheless, our uncontrolled study setting reflects reality: we implemented this pragmatic study at actual OPS, and enrolled real clients presenting to use drugs in their usual manner. Therefore, our results accurately reflect the complexity of both individuals’ drug use patterns and physiologic response to smoking opioids in real-world settings. For instance, we could not control the strength and duration of individual inhalations, and therefore, individual inhalations were highly variable and cumulative numbers of inhalations were not necessarily comparable. This reality reflects the challenge of studying this topic area among people who were exposed to unregulated drugs and who used them in variable ways, and the challenge of trying to understand risk factors for hypoxemia while people smoked opioids in real world, uncontrolled settings. Furthermore, implementation of our study at four select OPS in BC limits generalizability, as individual OPS vary in their clientele, available resources, physical layouts, and staff comfort and capacity, and there are regional variations in unregulated drug supply composition. Nonetheless, our engagement of peers to lead the research provides an accurate indication of how such a protocol would perform at actual peer-led OPS.

### Future directions

Our findings have broad applicability to supervised drug use sites internationally that monitor people while smoking opioids: continuous pulse oximetry should be piloted in those settings to understand unique implementation needs. Additionally, our continuous pulse oximetry protocol should be expanded to people who use drugs by other means (e.g., injection), to better understand how SpO_2_ changes in relation to drug use by other modalities in real world settings. Future studies should seek to capture more granular details about smoking (e.g., frequency, depth, and duration of inhalations) to more accurately model associations between dose of drug received by smoking and SpO_2_ response. Furthermore, studies should attempt to identify drug type and dose ingested through formal drug checking services at point of participation. Finally, qualitative studies should explore peoples’ experiences with overdoses while smoking opioids, to better understand the circumstances, behaviors, and risk factors associated with overdose.

## Conclusions

Our study indicates that continuous pulse oximetry is a safe adjunct for monitoring people who smoke opioids at OPS, and complements our feasibility analysis in which OPS staff and clients identified that SpO_2_ monitoring increased their sense of safety, comfort, and allowed physical distancing. Our data also reflect challenges of monitoring at real-world OPS sites, indicating that OPS that permit drug use by inhalation require greater supports to optimally monitor clients and implement monitoring technology. Our data demonstrated heterogeneity within and between participants’ SpO_2_ levels across monitoring events, indicating the complexity of SpO_2_ responses to smoking unregulated opioids in real-world settings. Although our data showed an association between six or more inhalations and probability of SpO_2_ drop, we did not find a consistent association between cumulative inhalations and hypoxemia beyond seven inhalations. There is a need to better understand the characteristics of smoking (e.g., frequency, depth, duration), participants (e.g., opioid tolerance), and drug use settings (e.g., using alone vs. in social settings, feeling “rushed”) that contribute to hypoxemia and overdose risk.

### Electronic supplementary material

Below is the link to the electronic supplementary material.

Supplementary Material 1

Supplementary Material 2

## Data Availability

The datasets used and analyzed during the current study are available from the corresponding author on reasonable request.

## Abbreviations

SpO_2_: oxygen saturation
OPS: overdose prevention services
BC: British Columbia
