# Supplementary material for: Physiologic oxygen responses to smoking opioids: an observational study using continuous pulse oximetry at overdose prevention services in British Columbia, Canada
Source: Harm Reduct J. 2024 May 3;21:89. doi: 10.1186/s12954-024-01011-z (PMC11067188; doi:10.1186/s12954-024-01011-z)
Supplement: Supplementary file 2 — Supplementary Material 2 [file 12954_2024_1011_MOESM2_ESM.doc]

**APPENDIX 2: STRUCTURED OBSERVATION FORM**

**Preventing Opioid Deaths due to COVID Related Increase in Smoking Illicit Substances (Preventing OD CRISIS)**

Incident Collection Form | Overdose Prevention Site

| Participant ID |  |  |
| --- | --- | --- |
| Start Time (iPad time 0) |  | **start stopwatch* |

1. **Time of inhalations**

| **Minute** |  |  |  |  |  |  |  |  |  |  |  |  |  |  |  |  |  |  |  |  |  |  |  |
| --- | --- | --- | --- | --- | --- | --- | --- | --- | --- | --- | --- | --- | --- | --- | --- | --- | --- | --- | --- | --- | --- | --- | --- |

1. **Time of alarms,** **interventions, and response**

| **Minute** |  |  |  |  |  |  |  |  |  |  |  |  |  |  |  |  |  |  |  |  |  |
| --- | --- | --- | --- | --- | --- | --- | --- | --- | --- | --- | --- | --- | --- | --- | --- | --- | --- | --- | --- | --- | --- |
| Alarm sounds |  |  |  |  |  |  |  |  |  |  |  |  |  |  |  |  |  |  |  |  |  |
| Staff responds to client |  |  |  |  |  |  |  |  |  |  |  |  |  |  |  |  |  |  |  |  |  |
| **Respiratory depression noted*** |  |  |  |  |  |  |  |  |  |  |  |  |  |  |  |  |  |  |  |  |  |
| **Overdose noted**** |  |  |  |  |  |  |  |  |  |  |  |  |  |  |  |  |  |  |  |  |  |
| Verbal stimulation |  |  |  |  |  |  |  |  |  |  |  |  |  |  |  |  |  |  |  |  |  |
| Physical stimulation |  |  |  |  |  |  |  |  |  |  |  |  |  |  |  |  |  |  |  |  |  |
| Intramuscular naloxone |  |  |  |  |  |  |  |  |  |  |  |  |  |  |  |  |  |  |  |  |  |
| Intranasal naloxone |  |  |  |  |  |  |  |  |  |  |  |  |  |  |  |  |  |  |  |  |  |
| Oxygen applied |  |  |  |  |  |  |  |  |  |  |  |  |  |  |  |  |  |  |  |  |  |
| Rescue breaths (mouth-to-mouth) |  |  |  |  |  |  |  |  |  |  |  |  |  |  |  |  |  |  |  |  |  |
| Breaths with bag valve mask |  |  |  |  |  |  |  |  |  |  |  |  |  |  |  |  |  |  |  |  |  |
| Chest compressions |  |  |  |  |  |  |  |  |  |  |  |  |  |  |  |  |  |  |  |  |  |
| Oral airway inserted |  |  |  |  |  |  |  |  |  |  |  |  |  |  |  |  |  |  |  |  |  |
| Nasal airway inserted |  |  |  |  |  |  |  |  |  |  |  |  |  |  |  |  |  |  |  |  |  |
| Automatic external defibrillator |  |  |  |  |  |  |  |  |  |  |  |  |  |  |  |  |  |  |  |  |  |
| Intramuscular epinephrine |  |  |  |  |  |  |  |  |  |  |  |  |  |  |  |  |  |  |  |  |  |
| **Client response***** |  |  |  |  |  |  |  |  |  |  |  |  |  |  |  |  |  |  |  |  |  |
| 9-1-1 called |  |  |  |  |  |  |  |  |  |  |  |  |  |  |  |  |  |  |  |  |  |
| Transported to hospital |  |  |  |  |  |  |  |  |  |  |  |  |  |  |  |  |  |  |  |  |  |

* Respiratory depression: slow breathing, <10 breaths per minute or <1 breath per 6 seconds

**Overdose: No response to verbal or physical stimulation *and* slow breathing (< 10 breaths per minute or <1 breath per 6 seconds)

***Response: client responds to verbal and/or physical stimulus, and respiratory rate increases to >10 breaths per minute
